# Strong coupling and vortexes assisted slow light in plasmonic chain-SOI waveguide systems

**DOI:** 10.1038/s41598-017-07700-z

**Published:** 2017-08-03

**Authors:** Giovanni Magno, Mickael Fevrier, Philippe Gogol, Abdelhanin Aassime, Alexandre Bondi, Robert Mégy, Béatrice Dagens

**Affiliations:** 1 Centre de Nanosciences et de Nanotechnologies, CNRS, Univ. Paris-Sud, Université Paris-Saclay, C2N – Orsay, 91405 Orsay cedex, France; 2 E.I.CESI, Campus Ide-de-France/Centre, 93 Bld de la Seine, BP 602-92006 Nanterre Cedex, France

## Abstract

A strong coupling regime is demonstrated at near infrared between metallic nanoparticle chains (MNP), supporting localized surface plasmons (LSP), and dielectric waveguides (DWGs) having different core materials. MNP chains are deposited on the top of these waveguides in such a way that the two guiding structures are in direct contact with each other. The strong coupling regime implies (i) a strong interpenetration of the bare modes forming two distinct supermodes and (ii) a large power overlap up to the impossibility to distinguish the power quota inside each bare structure. Additionally, since the system involves LSPs, (i) such a strong coupling occurs on a broad band and (ii) the peculiar vortex-like propagation mechanism of the optical power, supported by the MNP chain, leads to a regime where the light is slowed down over a wide wavelength range. Finally, the strong coupling allows the formation of guided supermodes in regions where the bare modes cannot be both guided at the same time. In other words, very high k modes can then be propagated in a dielectric photonic circuit thanks to hybridisation, leading to extremely concentrated propagating wave. Experimental work gives indirect proof of strong coupling regime whatever the waveguide core indexes.

## Introduction

Plasmonic surface polariton (SPP) waveguides have attracted an increasing attention in the past few years owing to their ability to confine light below the diffraction limit^1^, thereby potentially enabling device miniaturization at the nanoscale with dimensions not accessible with conventional dielectric waveguides. So far, several types of plasmonic waveguides supporting propagative surface plasmon polaritons have been reported, including long range surface plasmon polaritons^2, 3^, dielectric loaded waveguides^4, 5^, metal-insulator-metal slot waveguides^6^.

Metallic nanoparticle (MNP) chains, supporting localized surface plasmon (LSP) modes, can confine light at still smaller scales than the SPP systems^7–10^. Their resonances are very sensitive to the external environment thus opening the way toward bio- and chemical sensors^11, 12^. Energy exchange between an ensemble of coupled MNPs and a dielectric waveguide has been demonstrated by several authors using a free-space optical excitation of the MNP chain^13^. Optical propagation along a gold nanoparticle chain has been investigated in a guided configuration where the chain was excited through the evanescent field of a silicon-on-insulator (SOI) waveguide^14^. Dispersion curves of the MNP chain were thus explored outside the light cone, and results showed that the optical energy carried by the TE dielectric waveguide mode could be totally transferred into the transverse plasmon mode of the MNP chain. For a wide wavelength region around the LSP resonance, the system was found to behave as a coupled-waveguide system with an ultra-small coupling length between the two guides. Recently, MNP chain coupled with standard SOI waveguide has been successfully exploited to conceive very efficient integrated plasmonic tweezers, able to perform linear repositioning of a trapped nanoparticle thanks to the peculiar dispersion shown by this coupled system^15^.

Coupling phenomena between waveguides and/or resonant systems have generated an abundant literature during past decades^16–22^, but only a few papers are consecrated to the case of strong coupling regime^23–25^. Recently, strong coupling has been demonstrated between a surface plasmon propagating on a planar silver thin film and the lowest excited state of CdSe nanocrystals^26^. This regime has also been claimed for a coupled-waveguide system formed by SOI waveguides and a plasmonic nanogap supporting a propagative surface plasmon polariton^27, 28^.

As well known in quantum electrodynamics (QED) cavities, in lossy coupled systems two different regimes can be identified by the loss to coupling ratio. In simple words, coupling can be seen as the energy exchange between the systems, whereas losses accounts for energy dissipation. When the coupling dominates the losses, the so called strong coupling regime^29^ occurs and the whole system cannot longer be described as the superposition of the two original ones and their individuality is lost. A sufficient condition for strong coupling is the avoided resonance crossing entailing energy levels splitting^30^. When the anticrossing can be observed, in fact, the separation of the two involved energy levels has to be larger than the losses, which cause a spectral broadening of the branches involved^31^.

In this paper, we present a detailed investigation of the coupling regime in optical systems comprised of a dielectric waveguide having small intrinsic losses and a lossy metallic nanoparticle chain deposited on top of the waveguide. By considering two different core materials – a SOI waveguide with a high index core (n = 3.47) and a Si_3_N_4_ waveguide with a modest index core (n = 1.98), we show that the strong coupling occurs mainly thanks to the geometry and significantly modifies the propagation properties in a wide wavelength range. Recently, in Dagens *et al*.^32^, we reported on the experimental scanning near-field optical microscopy (SNOM) characterisation of the same SOI-based structure that will be described in this paper, demonstrating the excitation of two distinct supermode having distinct propagation constants (see Fig. 2 in ref. 32). In this contribution, we aim to numerically shed light on the underlying mechanisms of the strong coupling regimes. The intrinsic nature of the MNP waveguide allows the MNP chain to be in direct contact with the dielectric waveguide: the mode profiles of individual guiding structures strongly overlap with the consequence that the coupled system can no longer be described within a perturbative approach. Finally, two supermodes are clearly generated but they can no longer be recognized as a simple linear combination of the individual waveguides modes and the power flowing in one branch of the system cannot be unambiguously separated from that flowing in the other branch. More generally the characteristics of the both supermodes (losses, group velocities, …) become similar despite very different individual waveguides. In analogy with other coupled systems^26^, the difference between their propagation constants is expected to be of the same order of magnitude as the propagation constants themselves (∆*n*/*n* > 0.1). Following the improved coupled waveguide mode theory^25^, the power maximum in one waveguide is predicted not to spatially coincide with the power minimum in the other one.

All these features of the strong coupling regime are verified using 3D finite-difference time-domain (FDTD) simulations of the coupled systems and harmonic inversion of the spatial distribution of the fields (see the Methods﻿ section for details). Then, a detailed analysis of the supermodes dispersion and absorption, and of the intensity and field profiles of supermodes is carried out, and shows the vortexes assisted slow light imposed by the localized plasmonic resonance to the whole system thanks to the strong energy exchange. Finally, the involved structures are fabricated with long MNP chains comprised of 50 nanoparticles. A complementary waveguides transmission characterization confirms that plasmonic modes can be here efficiently excited well far from phase matching conditions.

## Results and Discussion

**Figure 1:**
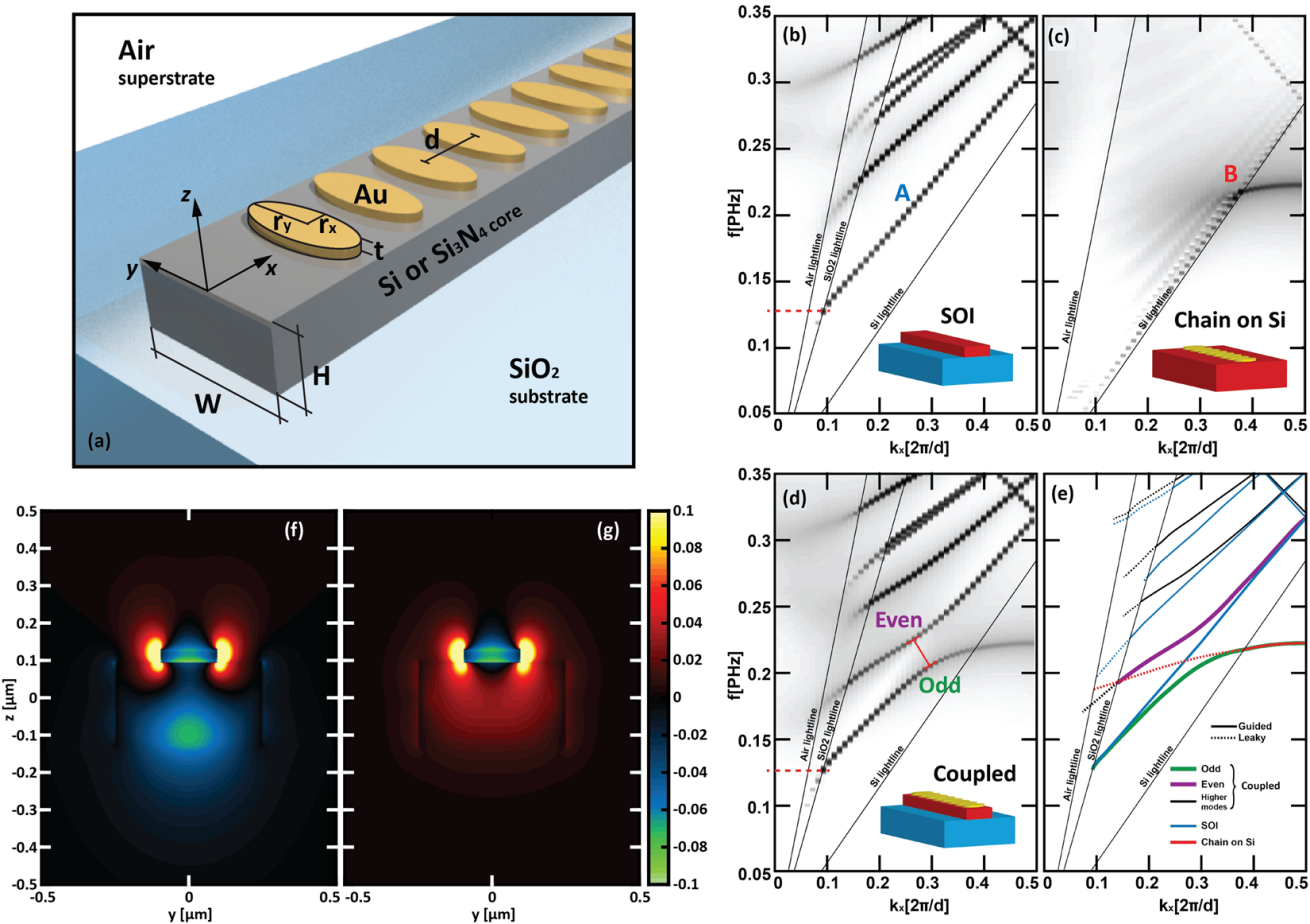
(**a**) Sketch of the proposed coupled system. (**b**–**e**) FDTD calculated TE band structures of a: (**b**) standard SOI waveguide; (**c**) infinite-long MNP chain placed on a semi-infinite Si substrate; (**d**) full coupled structure. (**e**) Hand-made tracing of the curve superposition. (**f**,**g**) Self-normalized *y*-component of the Electric field calculated on the coupled structure for *λ* = 1.450 *μm* (*f* = 0.207 PHz). In particular, the figure depicts the (**f**) even and the (**g**) odd supermode, calculated when (**f**) *k*_*x*_ = 0.2 *2π/d* and (**g**) *k*_*x*_ = 0.3 *2π/d*, respectively.

A schematic of the investigated structures is shown in Fig. 1(a). A traditional SOI waveguide, having a Si rectangular core, with height *H* and width *W* equal to 220 *nm* and 500 *nm* are placed on top of a *SiO*_*2*_ substrate^14^, constitutes the dielectric waveguide (DWG) of the coupled system. A linear chain of *N* gold ellipsoidal nanocylinders, having radii *r*_*x*_ and *r*_*y*_ equal to 42.5 *nm* and 100 *nm*, a height *t* = 30 *nm* and a period *d* = 150 *nm*, placed on top of the dielectric waveguide core, constitutes the localized surface plasmon mode waveguide (LSPWG). It is worth pointing out that, to provide a more realistic model of the natural oxidisation occurring in Si boundaries, a SiO_2_ thin layer, having a thickness of 2 nm, has been placed between the Si core and the gold particles. Later in this manuscript we will consider also a Si_3_N_4_ core having *H* and *W* equal to 500 *nm* and 1000 *nm*. In this case, the radii of the LSPWG elements are chosen equal to 152.5 *nm* and 63 *nm*, respectively, and their height equal to 65 *nm*. The ellipsoidal nanocylindric shape of the chain elements is chosen to enhance transverse dipole coupling along the chain at near infrared. The geometrical parameters are determined so as to operate in the wavelength region around 1400 *nm*. The structure has been modelled by considering dispersive and lossy material models which have been detailed in the Methods section of this paper.

### Supermodes analysis

Firstly, the coupling mechanism of an infinitely long coupled structure has been investigated by means of FDTD-calculated photonic band diagrams. Details about the calculations have been given in the Methods section. The same analysis has been performed also for the two uncoupled waveguides. Although it is possible to link the modal losses per unit length to the increased spectral linewidth of the mode through the group velocity^33^, the harmonic-inversion technique^34^ has been used instead for a more reliable calculation of modal losses. Thus, for the sake of a better readability of the band structures reported, we display the self-normalised logarithm of the power spectral density defined in Eq. (5) of the Methods section, since they are intended to be representative of the real part of the modes propagation constants.

Figure 1(b–e) shows the photonic band diagrams calculated for the coupled systems and for the bare waveguides. In particular, in Fig. 1(b–d) we display the TE band structures of the SOI waveguide, of an infinite-long MNP chain placed on a semi-infinite Si substrate and of the full coupled structure. In Fig. 1(e) a hand-made tracing of the curve superposition has been reported to ease the comparison.

In Fig. 1(b) it is possible to observe that, for wavelengths larger than 2.355 [*μm*] (indicated by the red dashed line), the fundamental TE mode of the SOI waveguide (labelled A) becomes to leak toward the SiO_2_ substrate. A second order leaky mode appears for wavelength between 1.480 *μm* and 1.316 *μm*, which becomes guided for shorter wavelengths. The band structure of the LSPWG, depicted in Fig. 1(c), demonstrates that an almost horizontal LSP-like guided mode (labelled B) exists between 1.350 *μm* (corresponding to a frequency *f* = 0.22 PHz) and 1.383 *μm* (below the substrate lightline). It becomes radiative (toward the substrate) for longer wavelengths and “smears out” as it approaches the superstrate lightline (see ref. 35 for further details).

As can be inspected from Fig. 1(d), a large anti-crossing opens between two dressed modes. This can be recognized as the effect of the strong coupling between two bare modes: the fundamental TE mode of the SOI waveguide (mode A, in Fig. 1(b)) and the LSP-like mode supported by the MNP chain placed on top of a Si substrate (mode B, in Fig. 1(c)). The leaky tail of mode B crosses the guided mode A and forms the even and odd supermodes of the full structure. It is worth pointing out that, in this system, the action of the strong coupling hybridises the dielectric and the LSP modes allowing the existence of the dressed modes as guided modes in regions where the bare modes cannot be both guided at the same time. In fact, for instance, the guided region for the bare LSPWG constitutes a forbidden region for the bare DWG. Vice versa, the leaky region of the bare LSPWG, between the Si and the SiO_2_ lightlines, corresponds to a guided region for the bare DWG. On the contrary, thanks to the hybridisation, the dressed modes are allowed to be both guided in the same region, as long as they are located below the SiO_2_ lightlines. Figure 1(f) and (g) display the profile of the Electric field (*y*-component) calculated over the elementary cell of the coupled structure along the [*y*, *z*] plane (which cuts the elementary cell along its centre with respect to the x direction) when *λ* = 1.450 *μm*. In particular, Fig. 1(f) and (g) show the odd and even supermodes appearing at about *k*_*x*_ = 0.2·*2π/d* and *k*_*x*_ = 0.3·*2π/d*, respectively. As can be noticed, the field profiles are quite “delocalized” and tangled so that the profiles of the uncoupled waveguide are hardly recognizable.

**Figure 2:**
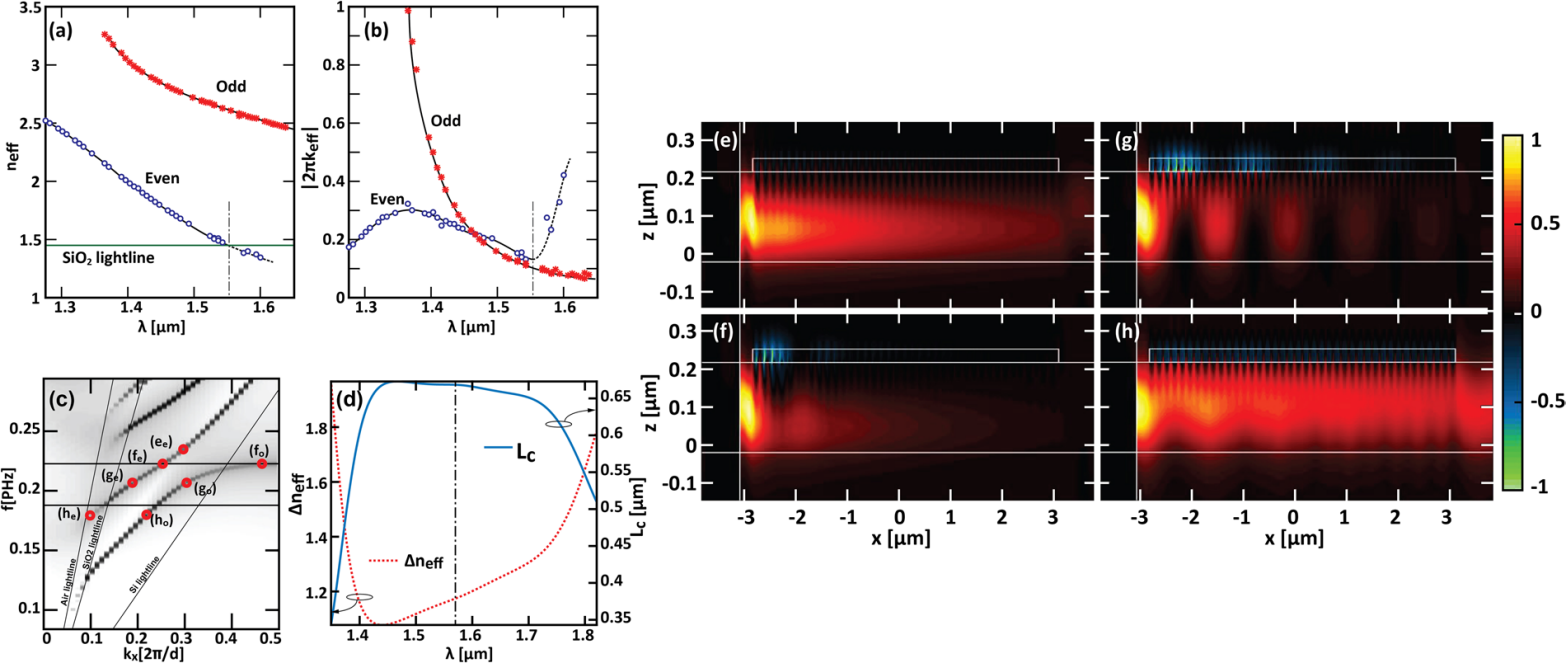
(**a**) Dispersion and (**b**) absorption curves when a coupler having 50 MNP is considered. In (**a**) the SiO_2_ substrate lightline is superimposed (green horizontal line). The vertical black dash-dotted line indicates the wavelength where the *n*_*eff*_ of the even mode crosses the aforementioned lightline. (**c**) Coupled device band structure. (**d**) Supermodes effective refractive index mismatch (red dotted curve) and corresponding coupling length (solid blue curve) calculated as a function of the wavelength (e-h) Self-normalized *y*-component of the Poynting vector for y = 0 calculated at (**e**) *λ* = 1.275 *μm* (*f* = 0.235 PHz), (**f**) *λ* = 1.350 *μm* (*f* = 0.22 PHz), (**g**) *λ* = 1.450 *μm* (*f* = 0.207 PHz) and (h) *λ* = 1.651 *μm* (*f* = 0.182 PHz). In (**c**) the frequencies corresponding to the distribution reported in (**e**–**h**) are highlighted by red circles. The two superimposed horizontal black lines delimit the zone where the two supermodes contemporarily exist as guided modes. These maps correspond to the states highlighted in (**c**) by red marks.

To further deepen the analysis, by using a well-established software for solving the problem of the harmonic inversion^34^, we have calculated the complex effective refractive index *n* = *n*_*eff*_ –*jk*_*eff*_ of both the supermodes, where its imaginary part *k*_*eff*_ accounts for their modal losses (see the Methods section for further details on this calculation). Figure 2(a) and (b) display the dispersion (n_eff_(f, k_x_)) and the absorption (2πk_eff_(f, k_x_), equal to λ time the attenuation constant α) curves for the finite coupled structure. As can be inspected by the Fig. 2(b), the absorption curves of the even and the odd supermodes cross in the vicinity of 1.450 *μm*, in the same range where the dispersion curves anticrossing occurs. In the crossing point k_eff_ is equal to 0.0363, corresponding to a modal propagation length of about 3.2 *µm*. This absorption curves crossing, which is a characteristic fingerprint of strong coupling regime^36, 37^, indicates a balanced loss sharing between the supermodes mediated by the energy bouncing between the two guiding structures.

### Power coupling and slow light

Energy exchange in this strongly coupled system involves a slow light waveguide (in the LSPWG) and leads to specific behaviours which can be further investigated. Figure 2(e–h) show the *y* = 0 cuts of the real part of the self-normalized Poynting vector *x*-component *S*_*x*_, for the 50 MNP long coupler, calculated at four different wavelengths. As briefly shown in ref. 14, depending on the wavelength, different coupling regime can be recognized. For shorter wavelengths than *λ* = 1.350 *μm*, only the even supermode exists and has a dispersion curve which closely resembles that of the DWG fundamental TE mode. In this region, the system behaves as a lossy “effective waveguide” (state e_e_ in Fig. 2(c)), which is excited by means of a butt-coupling mechanism from the input SOI waveguide (without MNPs): as can be seen in Fig. 2(e), at *λ* = 1.275 *μm*, the injected power propagates within the coupled system and decays with an exponential trend. As soon as we fall within the range of the odd supermode linewidth, the DWG and the LSPWG starts exchanging energy via the supermodes generation, with the losses of the odd supermode are much higher than those of the even one (see Fig. 2(f)).

In Fig. 2(f), where the structure is excited at *λ* = 1.350 *μm*, we can observe that, due to this losses discrepancy, the odd supermode (f_o_ in Fig. 2(c)), which here shows a very low group velocity and a wavevector below the Si light line, vanishes abruptly, leaving the sole even mode to propagate on the structure (f_e_ in Fig. 2(c)). For wavelength where the dressed modes losses are comparable, a coupled regime becomes observable, where the fingerprint of a clear-cut periodic energy transfer from the DWG to the LSPWG and *vice versa* can be clearly recognized. As depicted in Fig. 2(g), when λ = 1.450 *μm*, the two waveguides are *near*-*phase*-*matched*, and periodic oscillations are maintained over the whole device length (corresponding to the concurrent excitation and beating of the states g_e_ and g_o_ in Fig. 2(c)), although the field attenuation along the structures is due to metallic losses. In Fig. 2(d) the difference between the effective refractive indexes of the odd and the even supermodes are reported as well as the resulting coupling length, calculated on an infinite long structure using the well-established relation^16^, L_c_ = λ/[2(n_eff,odd_ − n_eff,even_)]. This analysis reveals that around 1.450 *μm* the longest coupling lengths (about 650 *nm*) occurs. This is in full agreement with the data obtained from the harmonic inversion analysis performed on the finite 50 MNP long device. A notable feature that emerges from this calculation is that the coupling length remains almost constant after reaching its maximum, as a consequence of the quasi-parallelism of the supermodes dispersion curves in this spectral range. This behaviour is helped by the very similar losses undergone by the both supermodes on this wavelength range (shown in Fig. 2(d)), due to the strong coupling induced efficient modes mixing. The superimposed vertical black dot-dashed line highlights the transition wavelength toward the leaky region (inside the SiO_2_ light cone) of the even supermode. In fact, for longer wavelength, as the even mode (h_e_ in Fig. 2(c)) leaks out, the pronounced coupling behaviour observed in Fig. 2(g) gives way to a less marked energy exchange (Fig. 2(h)) and to a predominance of the odd supermode (h_o_ in Fig. 2(c)).

**Figure 3:**
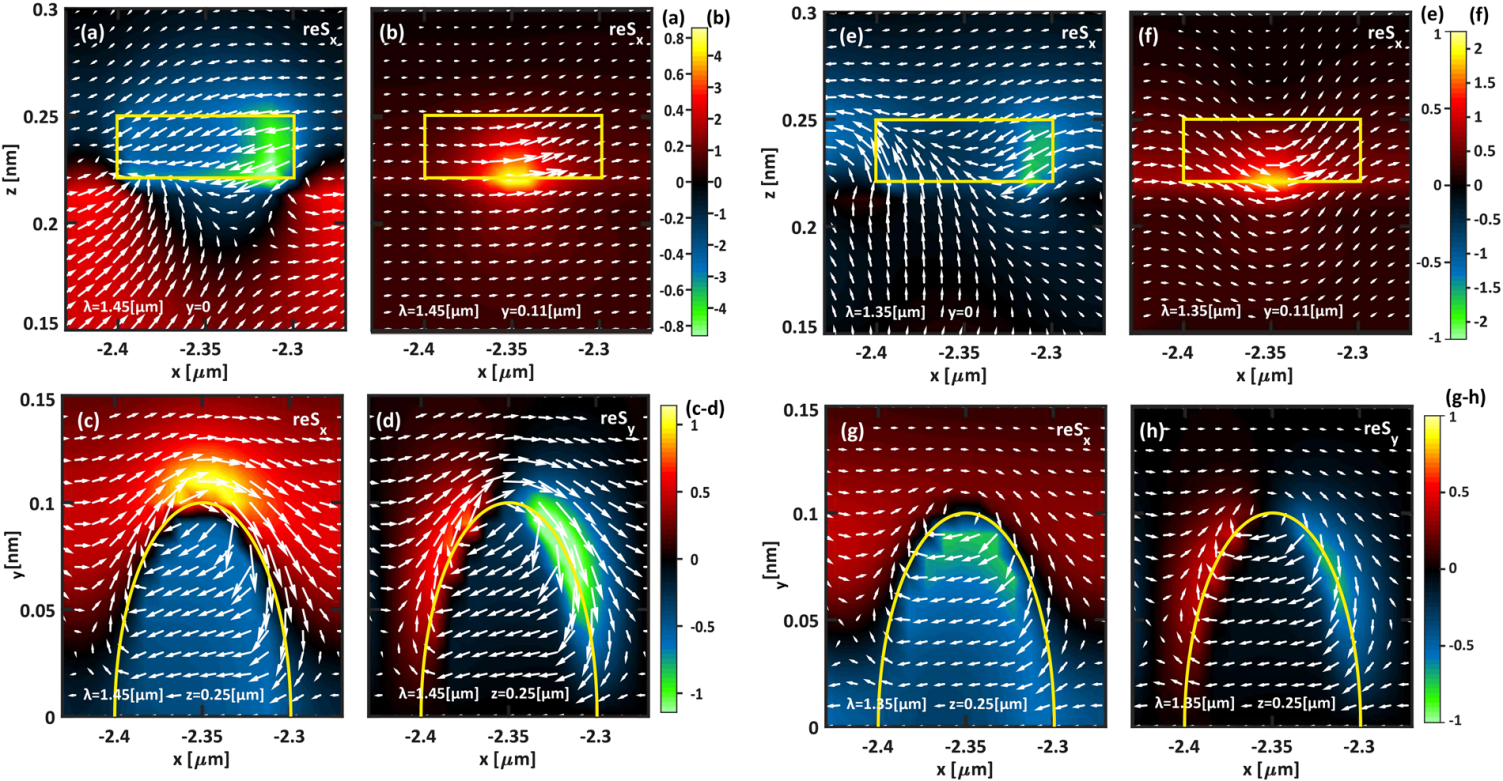
Poynting vector maps [arb. u.] and vector fields when: (**a**–**d**) *λ* = 1.450 *μm* and (**e**–**h**) *λ* = 1.350 *μm*. In (**a**),(**b**),(**e**),(**f**) the Poynting vector is plotted as a function of *x*- and *z*-coordinates, when: (**a**),(**e**) *y* = 0 and (**b**),(**f**) *y* = 110 *nm*. In (**c**),(**d**),(**g**),(**h**) the Poynting vector is plotted as a function of *x*- and y-coordinates, when *z* = 250 *nm* and *y* > 0. The yellow curves display the MNP contour with respect to the cut.

Additionally, the two quasiparallel supermodes dispersion curves, ranging from about λ = 1.350 *μm* to about λ = 1.651 *μm* (see the region between the points g and h of Fig. 2(c)), also present a reduced slope typical of slower light. Since the Poynting vector accounts for the direction of the energy flux, it can be used to analyse the local energy exchange in the system. For example another remarkable feature that stands out from Fig. 2(e–h) is that the power coupled on the LSPWG seems counter-propagative (see the sign of *S*_*x*_(*x*, *y* = *0*, *z*)). However, in a 3D structure, judging the overall direction of energy (and power) requires an observation of *S*_*x*_ in the whole space, and not only for *y* = 0. Figure 3 clarifies what actually happens by depicting different section of *S*_*x*_ within a close-up on one period of the LSPWG. In particular, Fig. 3(a) and (b) compare two *y*-section of *S*_*x*_ when *y* is equal to 0 and 110 *nm*, respectively, and when λ = 1.450 *μm*. To better visualize the power flow, the Poynting vector field arrows and the contour of the MNP across the section (see the superimposed yellow solid rectangle) are superimposed. Indeed in Fig. 3(a) the power seems counterpropagating across the LSPWG [*x*, *z*] plane of symmetry, showing weak counter clockwise vortexes located within the gap between two adjacent MNPs. However, as shown in Fig. 3(b), when *y* = 110 *nm* (in other words right alongside the MNPs chain) a co-propagating flow of the energy can be observed. To further shed light on this unusual behaviour, we show in Fig. 3(c) and (d) the *z*-cut of the real part of the *x*- and *y*-component of the Poynting vector, respectively, when *z* corresponds to the top facet of the MNPs. To better visualize the phenomenon and to provide a sufficient degree of magnification, we show in these cuts only one half of the particle (see the superimposed yellow solid contour. *S*_*x*_ and *S*_*y*_ are symmetric and antisymmetric, respectively, with respect to the *x*-axis). As it emerges from the observation of the superimposed Poynting vector field arrows, two contra-rotating energy flow vortexes appear in the proximity of the major axis extrema of the elliptic nanocylinders.

The overall effect of this behaviour is to strongly slow down the light as the odd supermode reaches its quasi-flat region near the band-edge of the first Brillouin zone. This becomes clearer by observing Fig. 3(e–h), showing the Poynting vector maps and vector fields when λ = 1.350 *μm*, where the odd supermode is close to the band-edge and has a marked LSPWG-like character.

In particular, the Poynting vector distribution shown in Fig. 3(g), due to the lack of the positive hotspots near the MNP tip observable in Fig. 3(c), suggest that here the co-propagating and contra-propagating power fluxes may cancel out. In order to prove this, we can exploit the equivalence between the energy velocity, accounted by the Poynting vector, and the group velocity, which holds within non-absorbing media^38^. Thus, let us define the positive and the negative parts of the x-component of the Poynting vector, accounting for the co-propagating and the contra-propagating power fluxes along the *x*-direction, as it follows:

1 $${S}_{+}^{MNP}=\{\begin{array}{cc}{S}_{x} & if\quad {S}_{x} > 0\\ 0 & elsewhere\end{array}\quad ,\quad {S}_{-}^{MNP}=\{\begin{array}{cc}{S}_{x} & if\quad {S}_{x} < 0\\ 0 & elsewhere\end{array}.$$

**Figure 4:**
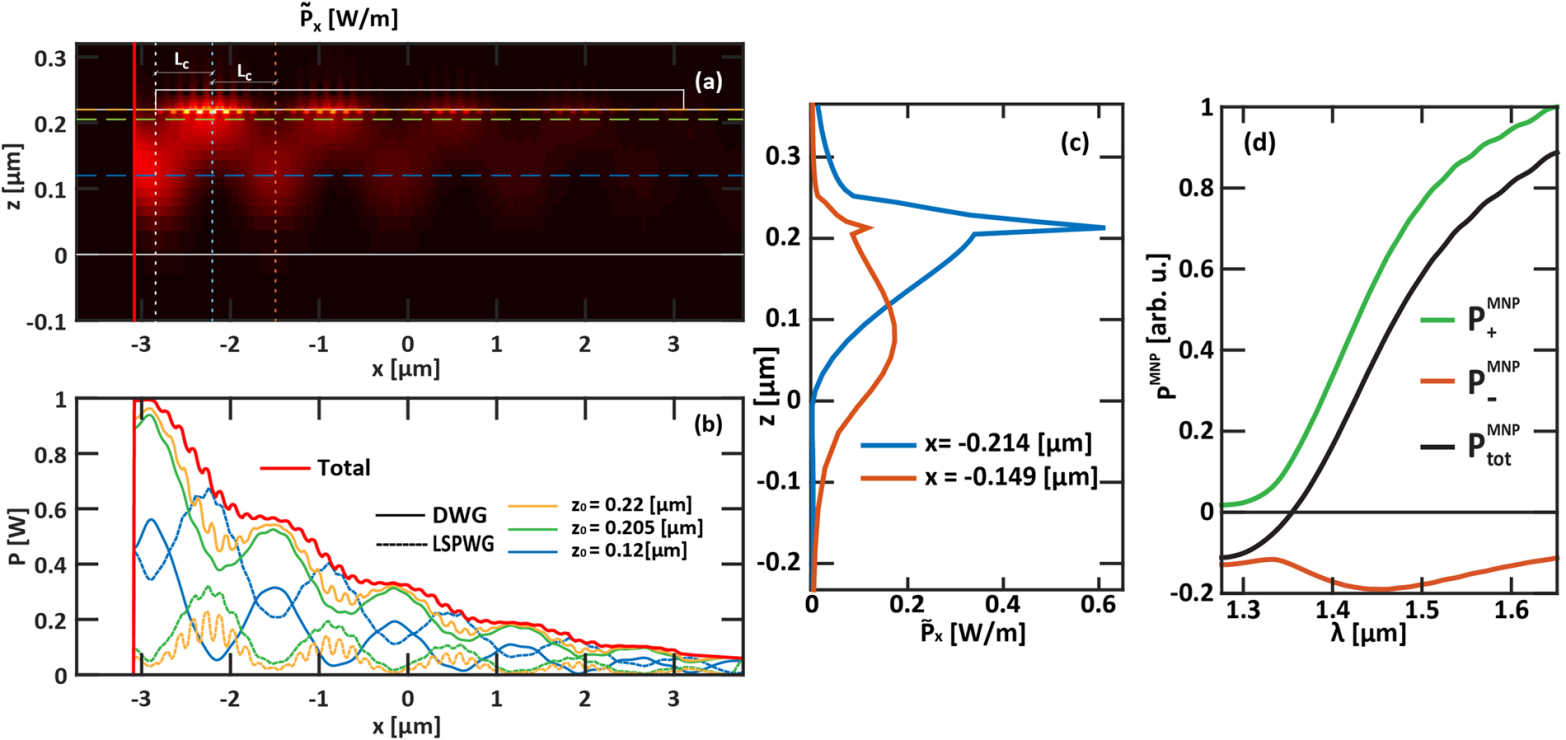
(**a**) $\tilde{{P}_{x}}$, defined in Eq. (2), as a function of the x- and the z-coordinates when λ = 1.450 *μm*. The superimposed white solid thin lines represent the structure contours, whereas the white, light blue and orange thin dotted vertical lines represent *x* = *x*_*0*_, *x* = *x*_*0*_ + *L*_*c*_ and *x* = *x*_*0*_ + 2*L*_*c*_, respectively. The superimposed horizontal orange, green and light blue thin dashed lines represent the integration limit *z*_*0*_ = 0.22, 0.205 and 0.12 *μm*, respectively. (**b**) Integrated power, flowing in the DWG (solid curves) and in the LSPWG (dashed curves), as a function of the *x*-coordinate, when the integration limit is assumed equal to *z*_*0*_ = 0.22 (yellow curves), 0.205 (green curves) and 0.12 μm (blue curves). The red solid curve represents the total power as a function of the x-coordinate. (**c**) *z*-profiles of $\,\tilde{{P}_{x}},$ integrated over one period along the x-direction, centered at x = *x*_*0*_ + *L*_*c*_ (blue solid curve) and *x* = *x*_*0*_ + 2*L*_*c*_ (orange solid curve). (**d**) Positive (green curve), negative (orange curve) and total (black curve) part of the Poynting vector, integrated in the hemispace above the MNPs chain.

By averaging each of them in the semi-space above the MNPs we obtain two new quantities depending on the wavelength, ${P}_{+}^{MNP}(\lambda )$ and ${P}_{-}^{MNP}(\lambda )$, accounting for the mean positive and negative power flux. In this non-absorbing region and in spectral region where the supermode splitting occurs, we can hypothesize the electromagnetic fields as being mainly governed by the LSPWG, showing only a minor influence from the DWG. These quantities, along with their sum ${P}_{tot}^{MNP}(\lambda )$, are depicted in Fig. 4(d). In this figure stands out that the two opposite energy fluxes compensate close to λ = 1.350 *μm*, where the odd supermodes exhibits a quasi-flat behaviour entailing for a strong slow light regime.

Due to this intricate mechanism, a more consistent overall depiction of the power exchange must be given by summing up the Poynting vector along the y-direction. Thus, we calculate the following integrated power density:

2 $${\tilde{P}}_{x}(x,z,\lambda )={\int }_{y}{\rm{Re}}\{{S}_{x}(x,y,z,\lambda )\}dy.$$

Figure 4(a) depicts this quantity calculated at λ = 1.450 *μm*, which confirms the co-propagating nature of the coupling. Fig. 4(c) shows two curves: (1) to account for the waveguide periodicity, the first one (blue solid curve) is obtained by averaging $\tilde{{P}_{x}}$ over one period of the LSPWG, centred at *x* = *x*_*0*_ + *L*_*c*_ = 650 *nm* (*x*_*0*_ represents the beginning of the MNPs chain), where the maximum of $\tilde{{P}_{x}}$ occurs; (2) the second one (dark orange solid curve) displays $\tilde{{P}_{x}}$ averaged over one period centered around *x* = *x*_*0*_ + 2*L*_*c*_ where the power is returned in the DWG. As a further fingerprint of strong coupling regime, the two profiles are very strongly overlapped (about the 76.4%, calculated using Eq. (6) reported in the Method section).

This entails that is it not possible to clearly define the right amount of power in one branch or in the other. Because of this impossibility to distinguish the power quota between the two branches, any other tentative of calculation remains unsatisfactory. In fact, Fig. 4(b) shows different unsatisfactory calculation of the power flowing in the two branches of the coupler at λ = 1.450 *μm*. Here, dotted and solid curves represent the power flowing in the DWG and in the LSPWG branches, respectively. These curves are obtained by the integration along the *z*-direction of the quantity $\tilde{{P}_{x}}$ in the two hemispaces delimited by the vertical ordinate z = z_0_, by changing the limit of the integration. In particular, (i) yellow, (ii) green and (iii) blue curves are obtained when z_0_ corresponds to (i) the interface between the Si core of the DWG and the MNPs chain, (ii) when z_0 _= 0.205 *μm* and (iii) when z_0_ corresponds to the crossing of the two profiles shown in Fig. 4(c), respectively. The red curve represents the total power flowing in the whole system as a function of the x-coordinate. These results are not a good representation of the right amount of power flowing in the two branches of the couplers. In fact, the representation (i) and (ii) fail estimating the power quota within the LSPWG and so the coupling efficiency *η*, which can be calculated, in analogy to Eq. (4) in ref. 22, as it follows:

3 $$\eta =\frac{100}{{P}_{S}d}{\int }_{-d/2}^{d/2}\int {\tilde{P}}_{x}({L}_{c}+\alpha ,z,{\lambda }_{0})dzd\alpha ,$$

where, *x*_*s*_ is the position of the source, *P*_*s*_ the power injected by the source and *d* the chain period. At λ = 1.450 *μm*, *η* is higher than 74.5%. Furthermore, the representation (iii) fails estimating the power within the DWG, which must be unitary at the input section.

Besides, another subtler signature of the strong coupling regime is also obvious in Fig. 4(a): the field intensity maximum (resp. minimum) in the MNPs chain is longitudinally (*x*−) shifted from the field intensity minimum (resp. maximum) in the DWG. This second feature is to be related to the strong influence of overlap integrals between uncoupled waveguide modes in the theoretical description of the coupled system (Eqs 8.4.15–16 in ref. 25).

Finally, a first conclusion can be extracted from these results: the strong energy exchange between the system modes allows the very efficient excitation of a regime where the both supermodes have similar properties like group velocity and losses. Since the carried energy propagates through vortexes movements near the tips of gold ellipsoidal nanocylinder constituting the LSPWG the both supermodes support slowed down light, with a prominent slow light (group velocity near to zero) accounted by the odd supermode near Brillouin zone border.

### Strong coupling and refractive index contrast

**Figure 5:**
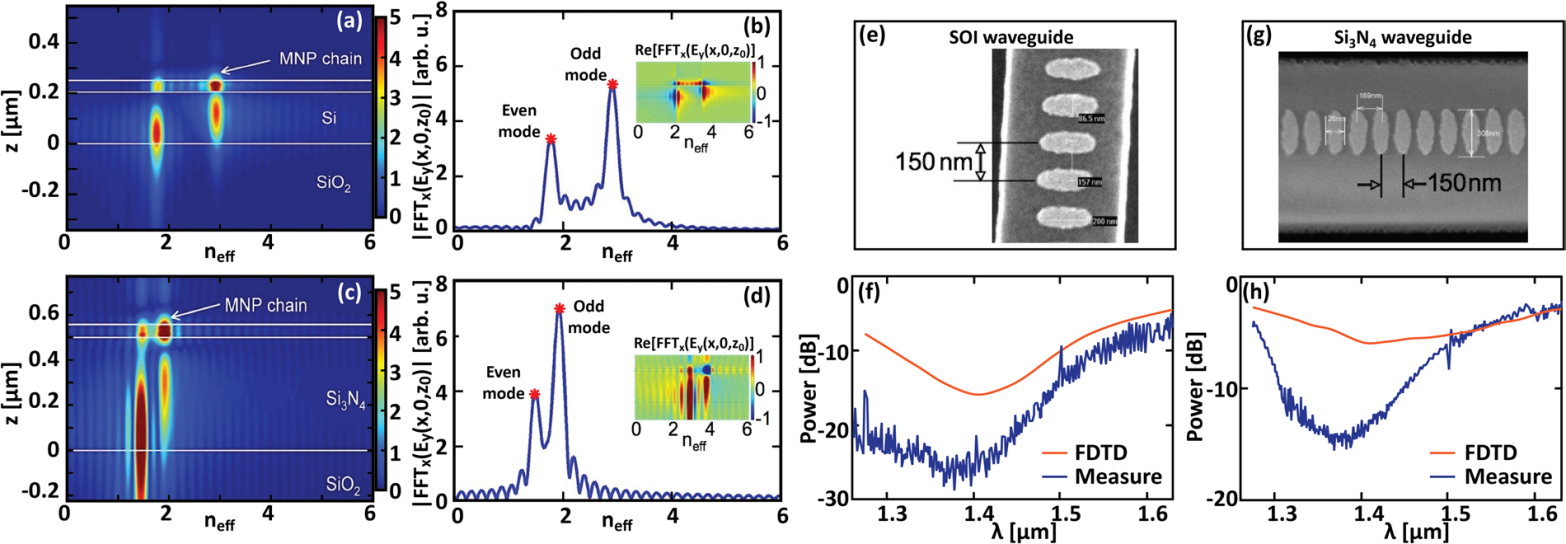
Spatial distributions and effective refractive indexes of supermodes revealed by the spatial Fast Fourier Transform (FFT) of the complex electric field E_y_. (**a**)(**c**) FFT module versus the effective index and the *z*-coordinate on in the [x, z] symmetry plane of the structure. (**b**),(**d**) FFT module calculated versus the effective index when *z* corresponds the LSPWG axis. Inset: real part of the FFT. In (**a**) and (**b**) a LSPWG/SOI system is considered, whereas (**c**) and (**d**) are calculated for the LSPWG/Si_3_N_4_ structure. (**e**) and (**g**) Scanning electron microscope images of gold nanoparticles deposited on top of SOI and Si_3_N_4_ waveguides, respectively. (**f**) Transmission spectrum measured (blue curve) and calculated (red curve) for a SOI waveguide with a chain of 50 gold nanoparticles. (**h**) Same as (**f**) for a Si_3_N_4_ waveguide with a chain of 50 gold nanoparticles.

A coupler having a Si_3_N_4_ core, with *H* and *W* equal to 500 *nm* and 1000 *nm*, is considered in order to reduce the index contrast at the two facets of the LSPWG. The MNP radii are chosen equal to 305 and 125 *nm*, respectively, to tune the resonance of the system at about 1,4 *µm*. In this case, the dispersion of the finite structure and the spatial distribution of supermodes of the coupled systems has been retrieved by means of an alternative way. From FDTD simulations on the full structure, we calculate the spatial Fast Fourier Transform (FFT) of the complex electric field *E*_*y*_. Fig. 5(c) and (d) show the FFT module versus the effective index n_eff_ and the *z*-coordinate in the [*x*, *z*] symmetry plane of the structure. For the sake of comparison, the same calculation has been also performed on the LSPWG/SOI device (reported in Fig. 5(a) and (b)). As confirmed by these maps, the even supermode (low-index mode) is essentially localized in the DWG while the odd supermode (high-index mode) is strongly confined in the LSPWG. For both systems (SOI and Si_3_N_4_), the even supermode is spatially shifted towards the lower cladding of the dielectric waveguide. The field maximum of the odd supermode is also slightly shifted towards the low index air medium.

Despite the fact that the difference between the effective indexes of supermodes is minimum at the phase matching wavelength, this difference is of the same amplitude as the effective indexes of uncoupled waveguides. For the LSPWG/SOI system, n_eff,even_ and n_eff,odd_ are respectively equal to 1.789 and 2.906 at λ = 1.450 *μm* (see Fig. 5(b)) while the effective index of the SOI waveguide mode is 2.512. For the LSPWG/Si_3_N_4_ system, the effective refractive index of the isolated Si_3_N_4_ waveguide mode is 1.623, while n_eff,even_ and n_eff,odd_ are respectively equal to 1.492 and 1.92 at λ = 1.400 *μm* (see. Fig. 5(d)), leading to a coupling lengths *L*_*c*_ = 1.635 *μm*. The relative variations of effective indexes ∆n_eff_/n_eff_ (of wavevectors, ∆k/k) are then found to be 0.44 and 0.26 for the LSPWG/SOI and LSPWG/Si_3_N_4_ systems, respectively. This is indeed a quantitative criterion for strong coupling. Thus, strong coupling in this system seems to be more driven by the structure geometry than the involved refractive indices or mode confinement.

Additionally, due to strong coupling regime, supermodes can be formed in non-guided ranges of the uncoupled system, whatever the waveguide core index, and even allows guiding of otherwise radiative modes. This is illustrated in Fig. 1(b–d) and Fig. 2(c) for SOI system where supermodes appear near the SiO_2_ light line (g_e_) and below the Si light line (f_o_). These supermodes present anyway high losses, as shown in Fig. 2(b).

### Experimental transmission spectra of coupled-waveguide systems

In ref. 32, we reported on the scanning near-field optical microscope (SNOM) characterisation of the same LSPWG/SOI structure here discussed. By Fourier transforming in space the complex near-field measured on the surface of the MNPs, we demonstrated that an even and an odd supermode appear with effective refractive index at 1.55 *µm* of about 1.6 and 2.7, respectively. These values are in good agreement with values numerically estimated in this contribution, corresponding to about 1.5 and 2.65 (at 1.55 *µm*). The slight difference can be most likely attributed to the fabrication tolerances, causing a slight spectral shift of the resonance.

Further, experiments were carried out to determine the transmission properties of LSPWG/SOI and LSPWG/Si_3_N_4_ coupled waveguide systems and show that supermodes can be excited far from phase matching conditions in these systems. Figure 5(e) and (g) show scanning electron microscope images of fabricated structures. Nanoparticles are precisely aligned along the axis of the dielectric waveguide. Their ellipsoidal shape is well verified. Residual irregularities, though small, are a little more pronounced in the Si_3_N_4_ case. Figure 5(f) and (h) show the measured transmission spectra of the waveguide structures and their comparison to simulation results. Transmission measurements are carried out by injecting a tunable TE polarized laser light through the input facet of the dielectric waveguide. The transmission spectrum is normalised by using a reference waveguide without nanoparticles on the same chip^14^.

As it is seen, measurement results are in agreement with numerical ones. The general shapes of transmission spectra as well as the transmission minima calculated from the FDTD model fit to those measured in the experiments for both systems. The lower transmission level measured in experiments (~−7 *dB*) is likely due to size fluctuations and shape irregularities of nanoparticles leading in turn to additional scattering losses. The small wavelength shift of the transmission minimum in the LSPWG/Si_3_N_4_ case (~20 *nm*) is attributed to slight deviations of the dielectric waveguide parameters from original specifications. The apparent “noise” in Fig. 5(f) (blue curve) is the consequence of parasitic reflections at the uncoated Si waveguide facets. Because of the lower index of silicon nitride, such reflections have a smaller influence for the LSPWG/Si_3_N_4_ system. In addition to the overall agreement between experiments and simulations, one important result of Fig. 5(h) is the higher transmission level obtained for the LSPWG/Si_3_N_4_ system. This reflects a weaker confinement of the odd supermode in the LSPWG and thus smaller propagation losses along the structure.

Finally, it is worthwhile noticing that the transmission spectra accounts for several band structure signatures. In particular, by observing both the calculated and measured transmittances of the LSPWG/SOI system, a slope change can be observed near λ = 1570 nm which can be addressed to the leaky-to-guided transition of the even supermode mode. For lower wavelengths, the transmission minimum is reached at about 1400 nm, which is near the transition of the odd mode to a deep slow light regime (the corresponding dispersion curve crosses the silicon lightline, as can be seen in Fig. 1(d)). A new slope change occurs around λ = 1350 nm, corresponding to the flat top region of the odd supermode dispersion curve and to the transition toward a faster light (and lower losses) regime of the even mode. Analogous considerations can be done on the transmittance of the LSPWG/Si_3_N_4_ case.

## Conclusions

In conclusion, the regime of strong coupling has been demonstrated for optical systems comprised of a dielectric waveguide and a gold elliptic nanocylinder chain supporting localized surface plasmons guided modes. The demonstration has been primarily carried out using FDTD simulations with an additional illustration from transmission measurements. Many features of the strong coupling regime have been illustrated for LSPWG/SOI system at telecommunication wavelengths. Supermodes formation has been also demonstrated for LSPWG/Si3N4 system, having a very different refractive index of the core materials with respect to the SOI-based one. Strong coupling is caused by the strong overlapping between modes of the individual guiding structures, which are in direct contact with each other by construction. This allows the fundamental TE mode of the DWG to excite the LSP-like mode of the LSPWG. The latter becomes radiative toward the DWG core, which, in turn, re-confines it as a guided mode. Such a waveguide configuration is unique in the sense that the plasmonic guide is directly deposited on the dielectric waveguide core and then forms a “cladding layer” of this waveguide. Energy exchange between the bare structures, generating the strongly coupled supermodes, have several consequences: (1) it is possible to find a regime where the vortexes assisted slow odd mode compensates for the propagative energy and vanishes overall energy transmission. Light is also slowed down in the wide wavelength range of strong coupling regime; (2) supermodes can be formed in non-guided or forbidden ranges of the bare constituent systems, whatever the waveguide core index; this implies that (2a) the hybridisation of the bare modes can modify the radiative character of the bare LSPWG and even (2b) extend the existence of the bare DWG mode within a region where propagation is forbidden (below the DWG core lightline); (3) it enables the existence of extremely concentrated and propagating waves. Present results can be generalized to similar systems with other materials at other wavelengths. In the near- and mid-infrared domains, strong coupling between a dielectric waveguide and a chain of metallic resonators forming a metasurface can be used to achieve compact devices with new functionalities in guided optics^12, 15^.

## Methods

FDTD modelling - In this paper, the numerical simulations are obtained by means of a fully 3D FDTD method technique (Lumerical FDTD Solutions). In all the simulations, gold complex permittivity ε_Au_ has been modelled by a Drude model fitting ellipsometric data in the infrared spectral region as it follows:

4 $${\varepsilon }_{Au}(\omega )={\varepsilon }_{\infty }-\frac{{\omega }_{P}^{2}}{\omega (\omega +j\gamma )},$$

where *ω* = 2*πf*, *f* is the frequency, ε_∞_ is the high-frequency permittivity (for ω → ∞), ω_P_ is the plasma frequency and γ is the collision frequency. In particular, we considered ε_∞_ = 1, ω_P_ = 1.29 × 10^16^ *rad/s* and γ = 6.478 × 10^13^ *rad/s*. The Si and the SiO_2_ have been modelled by fitting the dispersive models reported in ref. 39. The Si_3_N_4_ has been assumed non-dispersive in the spectral range of interest and modelled by a real refractive index of 1.98. The computational cell has been discretised by using a variable mesh requiring at least 34 mesh cells per wavelength. A fine uniform mesh region, with a resolution of 3 × 3 × 3 nm^3^ have been added around the LSPWG. A further mesh refinement has been used when the thin SiO_2_ oxidisation layer is considered, with a resolution of 1 nm in the direction of the layer thickness. The grading of the variable mesh has been set up in order to have a maximum growing rate of $\sqrt{2}$. Finally, boundary conditions and the size of the computational cell have been chosen accordingly to the specific calculation performed.

### Band structure calculation

In order to calculate the band structures reported in this paper, we used a set of M = 10 point-like monitors. For each of them we retrieved the electric field in the time domain. These monitors are randomly placed in the unitary cell of the periodic structures, within a region close to the metal nanoparticle. The unitary cell is excited by placing 5 electric dipoles, having random positions, orientations and initial phases, within the SOI waveguide core (or spanning along the whole cell when no SOI waveguide is considered). We require the sources to have a very narrow Gaussian temporal envelope, capable to provide excitation along the whole spectral range of interest (0.05 to 0.35 PHz). Along the x and the y directions, the computational cell is terminated by a couple of periodic boundary condition. Along the z-direction, the computational cell is terminated on perfect matched layers (PMLs).

For a given wavevector k_x_, for each point-like monitor *m* (=1..M) and for each component *s* (=x, y, z) of the electric field, the time signals e_ms_(t) is retrieved and the corresponding Fourier transform E_ms_(f) is calculated. Finally, the total power spectral density, recorded by the point-like monitors, is defined as:

5 $$PD({k}_{x},f)=\sum _{m=1}^{M}\sum _{s=x,y,z}{|{E}_{ms}(f)|}^{2}.$$

To retrieve the photonic band structure of the infinite long coupled system, PD(k_x_, f) is calculated for each k_x_-vector component within the positive half of first Brillouin zone (*k*_*x*_ ∈ [0, π/*d*]), which has been evenly discretised in a set of 50 points. It is worth pointing out that k_x_ represents the phase correction term of the fields at the boundary of the computational cell, along the x-direction. For the sake of readability, in Fig. 1(b,c,d) and in Fig. 2(c) we display the logarithm of PD(k_x_, f). Here, the resonant peaks correspond to the establishment of an optical mode. It is worth pointing out that, for the sake of simplicity and clarity, the calculation have been performed by imposing an antisymmetric boundary condition in the plane [*x*, *z*] for *y* = 0. This allows to cancel out any modes having a TM-like symmetry.

### Harmonic inversion-based modal losses calculation

For this purpose, we have performed FDTD simulation on the full structure, by considering a chain of 50 MNPs. A broadband source, having a spatial distribution matching the fundamental TE mode (*E*_*y*_ ≠ 0), is injected in the SOI waveguide and the whole electromagnetic field has been calculated by means of 3D volumetric monitors. We retrieve the complex electric field *E*_*y*_(*x*, *y* = *0*, *z*_*0*_) for *z*_*0*_ corresponding to the interface between the waveguide core and the MNP chain. Then, by using a well-established free software (Harminv^40^) for solving the problem of the harmonic inversion^34^, we calculate the complex effective refractive indexes *n* = *n*_*eff*_  − *jk*_*eff*_ of the existing modes from the electric field spatial distribution. In particular, modal losses per unit length can be retrieved as −(2πk_eff_/λ)∙(20Log_10_(e)) [*dB/m*].

### Evaluation of the power profiles overlapping

The overlap of the power profiles is evaluated as it follows:

6 $$100\frac{C(0)}{\mathop{{\rm{\max }}}\limits_{s}\{C(s)\}},$$

where C(s) represents the correlation integral. The latter is defined as:

7 $$C(s)=\int A(z)B(z+s)dz,$$

where A(z) and B(z) represent the two power distributions depicted in Fig. 4(b). C(s) describes the overlap integral for every possible relative shifting *s* of the two profiles A(z) and B(z).

### Samples fabrication

The fabrication of waveguide structures investigated in this work included several steps starting from SiO_2_/Si or SiO_2_/Si_3_N_4_ layers on silicon. The waveguide core was fabricated by using either deep UV (SiO_2_/Si_3_N_4_ system) or e-beam lithography (SiO_2_/Si system) followed by an etching process of the core layer. Structuring of gold nanoparticles was achieved by using e-beam lithography followed by a lift-off process. The lifted layers consisted of a 1 *nm* titanium adhesion layer and of a gold layer having a thickness of 30 nm and of 65 nm for the SiO_2_/Si and the SiO_2_/Si_3_N_4_ systems, respectively.
